# Western Spruce Budworm Outbreaks Did Not Increase Fire Risk over the Last Three Centuries: A Dendrochronological Analysis of Inter-Disturbance Synergism

**DOI:** 10.1371/journal.pone.0114282

**Published:** 2014-12-19

**Authors:** Aquila Flower, Daniel G. Gavin, Emily K. Heyerdahl, Russell A. Parsons, Gregory M. Cohn

**Affiliations:** 1 University of Oregon: Department of Geography, 1251 University of Oregon, Eugene, OR 97403-1251, United States of America; 2 Rocky Mountain Research Station: U.S. Department of Agriculture Forest Service, Rocky Mountain Research Station, 5775 U.S. West Highway 10, Missoula, MT 59808, United States of America; Ecole Pratique des Hautes Etudes, France

## Abstract

Insect outbreaks are often assumed to increase the severity or probability of fire occurrence through increased fuel availability, while fires may in turn alter susceptibility of forests to subsequent insect outbreaks through changes in the spatial distribution of suitable host trees. However, little is actually known about the potential synergisms between these natural disturbances. Assessing inter-disturbance synergism is challenging due to the short length of historical records and the confounding influences of land use and climate changes on natural disturbance dynamics. We used dendrochronological methods to reconstruct defoliator outbreaks and fire occurrence at ten sites along a longitudinal transect running from central Oregon to western Montana. We assessed synergism between disturbance types, analyzed long-term changes in disturbance dynamics, and compared these disturbance histories with dendroclimatological moisture availability records to quantify the influence of moisture availability on disturbances. After approximately 1890, fires were largely absent and defoliator outbreaks became longer-lasting, more frequent, and more synchronous at our sites. Fires were more likely to occur during warm-dry years, while outbreaks were most likely to begin near the end of warm-dry periods. Our results show no discernible impact of defoliation events on subsequent fire risk. Any effect from the addition of fuels during defoliation events appears to be too small to detect given the overriding influence of climatic variability. We therefore propose that if there is any relationship between the two disturbances, it is a subtle synergistic relationship wherein climate determines the probability of occurrence of each disturbance type, and each disturbance type damps the severity, but does not alter the probability of occurrence, of the other disturbance type over long time scales. Although both disturbance types may increase in frequency or extent in response to future warming, our records show no precedent that western spruce budworm outbreaks will increase future fire risk.

## Introduction

Natural disturbances can affect ecosystems in complex and often synergistic ways by changing their susceptibility to subsequent disturbances [1], [2]. A detailed understanding of synergism among natural disturbances is crucial for managing forests in the face of climate change and evolving land-use patterns. In forested ecosystems, insect outbreaks have long been assumed to increase the severity or probability of occurrence of fire through increased fuel availability, while fires may in turn alter susceptibility of forests to subsequent insect outbreaks through changes in the spatial distribution and density of suitable host trees [3], [4], [5]. In the interior Pacific Northwest, many Douglas-fir (Pseudotsuga menziesii (Mirb.) Franco) forests historically have been shaped by a combination of insect outbreaks and mixed-severity fires [6], [7], [8] suggesting the potential for synergistic interactions.

Douglas-fir forests are widespread in the interior Pacific Northwest, but their historical fire regimes have not been studied as intensively as many other forest types. Existing evidence indicates that these forests were historically characterized by a complex, spatially variable mix of fire return intervals and severities [6], [9], [10]. Relatively xeric Douglas-fir forests at lower elevations or on warm-dry aspects generally sustained high frequency, low severity fire regimes with low mortality among mature trees [6], [9]. In contrast, mesic Douglas-fir forests likely sustained fires less often and those fires were a patchy mosaic of low severity surface fires mixed with areas of stand-replacing crown fires [9], [8].

One of the most influential insects in Douglas-fir forests is the western spruce budworm (*Choristoneura occidentalis* Freeman). This species is a native lepidopteran defoliator that feeds primarily on Douglas-fir, grand fir (*Abies grandis* (Dougl. ex D. Don) Lindl.) and white fir (*Abies concolor* (Gord. & Glend.) Lindl. ex Hildebr.) trees. Western spruce budworm outbreaks may occur simultaneously across millions of hectares and often continue for a decade or more [11], [12], [13], [14], [15]. Defoliation by this species leads to reduced growth of host trees, and severe defoliation often leads to mortality of limbs or entire trees [16], [17], [18]. Defoliation severity is highly variable, with reported averages from 25% to 84% reduction in foliage [16], [19], [20]. Host tree mortality rates are also spatially variable at both stand and landscape scales and can vary both among and within host species. Average host tree mortality rates are typically below 10%, though stands with upwards of 30% mortality have been reported [21], [19]. Most host tree mortality is restricted to saplings and seedlings [16], [17], [19].

There is widespread speculation that the buildup of dead fuel during western spruce budworm outbreaks may increase future fire risk and/or severity in affected forests [22], [23], [14], yet few studies have assessed the spatial and temporal association between fires and western spruce budworm activity. The only studies to explicitly assess the statistical relationship between fire and western spruce budworm outbreak records reported a negative correlation between the disturbance types over a 3 to 6 year [24], [25]. However, these studies examined outbreaks solely during the late 20^th^ century when fires were being actively suppressed [24], [25].

Recent studies examining other insect species have found that the observed effect of insect activity on subsequent fire behavior is highly dependent on time-since-outbreak and weather conditions [26]. For example, there is a growing awareness that a positive association between bark beetle outbreaks and fire risk is limited to the earliest stages of an outbreak [27], [28], [26], [29], [30], [31]. Later in an outbreak cycle, the net effect of bark beetle induced mortality may actually be a damping of subsequent fire risk through the removal of fine fuels [32], [33]. Similar influences on fire have been observed or postulated for defoliating insects. Outbreaks of the eastern spruce budworm (Choristoneura fumiferana (Clemens)) may increase the probability of fire occurrence, but only during a narrow temporal window of 3–9 years after an outbreak [34], [35]. Conversely, fires may synergistically influence insect outbreaks as well. The effect of fires on subsequent insect outbreaks varies depending on insect species and feeding guilds. Fires are believed to increase susceptibility of trees to bark beetle-induced mortality by weakening their ability to resist attack [4], [5], [36]. In the case of defoliating insects, in the short term fire is more likely to decrease the susceptibility of forests to defoliator outbreaks by reducing the amount of foliage available for consumption [4], [5], [37]. These studies suggest that multi-directional synergistic relationships could occur between fire and western spruce budworm outbreaks.

Understanding synergism among natural disturbances requires that we also understand the role of climate and land use impacts in regulating natural disturbances so that we can distinguish apparent synchrony of disturbances caused by a common response to the same external forcing mechanism from synergism of disturbances caused by one disturbance type altering the probability of another disturbance type occurring. Changes in land use and climate have altered forest structure and disturbance regimes in western North America over the past century. Logging, intensive livestock grazing, and active fire suppression have increased the extent and homogeneity of dense forests of Douglas-fir, grand fir, and white fir, which are favored by the western spruce budworm and are, at least as saplings, less resistant to fire than ponderosa pines [38], [7], [39], [40]. At the same time, climate has changed over the 20^th^ century, with temperatures increasing across the Pacific Northwest [41]. Natural disturbance dynamics have changed in response to this combination of climatic and anthropogenic factors. The increased density of suitable host trees may be causing more severe, long-lasting, frequent, and synchronous outbreaks than those during previous centuries, as suggested by dendrochronological reconstructions in Montana, Oregon, Colorado, and New Mexico [42], [11], [12], [13]. Fire frequency was lower over the 20^th^ century than during previous centuries [43], though an increase in the spatial extent, and in some cases severity, of individual wildfires has also been observed over the last two-to-four decades in many forest ecosystems [9], [44], [45].

The short historical observational record unfortunately includes too few fires and outbreaks in western North America to allow a robust analysis of these complex ecological interactions. In particular, the historical record of budworm-fire interaction is strongly overprinted by land-use and fire-suppression effects, such that restoration of dynamics representative of less modified landscapes requires a paleoecological approach [46]. While fires [47], [6], [48], [49], [50] and western spruce budworm outbreaks [42], [13], [51] have been reconstructed using dendrochronological methods in mixed-conifer forests of the Pacific Northwest, they have rarely been jointly reconstructed at the same site. In previous studies in which both western spruce budworm outbreaks and fires were reconstructed for the same region using dendrochronological methods, the authors did not explicitly analyze or quantify disturbance interactions, and no obvious association between the two disturbances was apparent [42], [14]. Although both fires and western spruce budworm outbreaks can sometimes occur synchronously over large areas, detecting synergism between the two disturbance types requires analysis at the stand scale at which disturbances affect fuel characteristics and forest structure.

In this study, we used a combination of existing dendrochronological records of fires [52], [53] and western spruce budworm outbreaks [15] and eight new fire chronologies to compare paired disturbance histories at ten sites in Oregon, Idaho, and Montana. We used this multi-century dataset to answer the following questions: 1) have the frequency and duration of western spruce budworm outbreaks and fire changed over the last three centuries? 2) What is the relationship between moisture availability and the occurrence of western spruce budworm outbreaks and fires? 3) To what degree do western spruce budworm outbreaks and fires occur synchronously, asynchronously, or independently?

## Methods

### Study region

**Figure 1:**
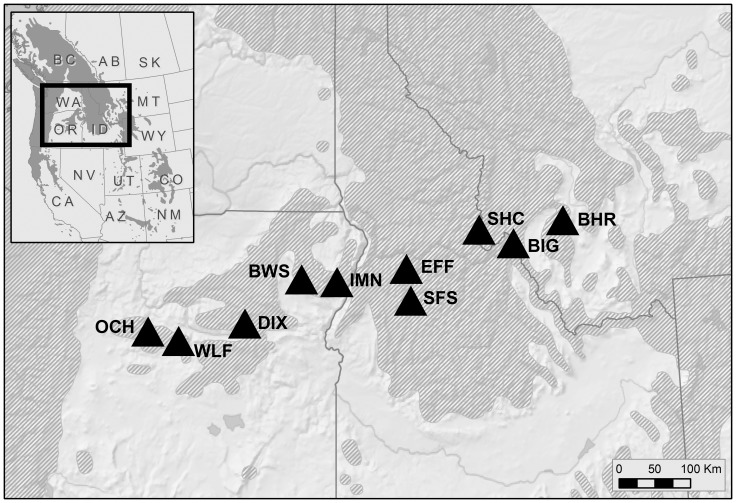
Location of sites at which we compared fire and western spruce budworm outbreak histories. Shaded area shows the distribution of Douglas-fir and grand fir (the primary host species for western spruce budworm) according to Little (1971) [86].

We analyzed fire and insect outbreak chronologies at 10 sites along a 600 kilometer transect from central Oregon to western Montana, spanning most of the longitudinal extent of mixed-conifer forests in the interior Pacific Northwest (Fig. 1; Table 1). We chose individual sites based on the presence of relatively old trees Douglas-fir and grand fir trees, which are the species preferred by western spruce budworm (i.e., “host” trees); visual or written evidence of recent western spruce budworm outbreaks; visibly fire-scarred trees (live or dead); and, where possible, the absence of logging or other direct anthropogenic disturbances. The sites are all in mixed-conifer forests dominated by a combination of Douglas-fir, ponderosa pine (Pinus ponderosa Dougl. ex Laws.), white fir and/or grand fir. Grand fir and white fir are often difficult to distinguish and are known to hybridize in some areas [54]. We therefore report white fir and grand fir together and, because grand fir is more common overall in our study area, will hereafter refer to them as grand fir. Douglas-fir trees accounted for over half of the total stand basal area among mature trees (i.e., with a diameter at breast height (DBH) of more than 15 cm) at eight sites. Some sites also had minor amounts of lodgepole pine (Pinus contorta Dougl. ex Loud.), western larch (Larix occidentalis Nutt.), western juniper (Juniperus occidentalis Hook.), or Rocky Mountain juniper (Juniperus scopulorum Sarg.). Density of mature (DBH>15 cm) trees was between 177 and 375 stems/ha.

**Table 1:** Site locations and stand characteristics.

Site	Latitude	Longitude	Elevation (m asl)	Aspect	Density (stems/ha)	Basal area (m^2^/ha)	Composition (%)			
							ABGR	PIPO	PSME	Other
OCH	44.45	−120.32	1700	WSW-SSW	333	104	30	11	52	7
WLF	44.34	−119.78	1755	S	343	85	1	41	58	0
DIX	44.58	−118.63	1660	S	354	40	66	21	3	11
BWS	45.15	−117.63	1550	SSW-SSE	177	66	11	15	74	0
IMN	45.12	−117.00	1385	S-E	313	49	50	40	10	0
EFF	45.39	−115.96	1670	W-WNW	292	82	2	3	95	0
SFS	44.87	−115.70	1283	WSW-WNW	188	26	0	45	55	0
SHC	45.75	−114.45	1603	SW	375	31	0	17	83	0
BIG	45.56	−113.84	1678	SW-WSW	313	51	0	28	72	0
BHR	45.82	−112.94	1930	WNW-WSW	302	32	0	0	99	1
**Average:**					299	56.6	16	22.1	60.1	1.9

Stand characteristics reported for trees with a diameter at breast height of at least 15 cm. Basal area is the total basal area of tree stems in the stand (m^2^/ha). Composition is reported as percent of total stand basal area. Species are as follows: ABGR =  Abies grandis, PIPO =  Pinus ponderosa, PSME =  Pseudotsuga menziesii, “Other”  =  Pinus contorta, Larix occidentalis, and/or Juniperus scopulorum.

Sites ranged in elevation from 1283 to 1930 m a.s.l. and had generally southerly or westerly aspects. The climate at these sites is continental with cold winters and warm summers. The sites are located in Oregon climate divisions 7 or 8, Idaho climate division 4, or Montana climate divisions 1 or 2. Based on the 1971 to 2000 climate normal PRISM data [55] downscaled using ClimateWNA software [56], our study area has an average annual temperature of 5.4°C (range: 4.1 to 7.2°C), an average January temperature of −4.3°C (range: −7 to −1.6°C), and an average July temperature of 16.1°C (range: 15.1 to 17.5°C). Average annual precipitation is 80.6 cm (range: 45.7 to 124.8 cm).

### Stand-level disturbance history reconstructions

We used a combination of existing dendrochronological records of fires [52], [53] and western spruce budworm outbreaks [15] and eight new fire chronologies to compare paired disturbance histories at 10 sites. We recorded location, elevation, slope, aspect, and forest composition at each site. To assess forest composition, we noted the species and DBH (at 1.4 m above the ground) of all “mature” (i.e., with a DBH>15 cm) trees within a 120×8 meter linear belt transect at each site. Transects were randomly placed within a stand known to contain both fire-scarred trees and host trees and roughly followed the slope contour.

We used well-established methods to reconstruct annually-resolved records of fire dates using samples from fire-scarred trees [57]. We used a chain saw to collect partial cross sections from three to eleven visibly fire-scarred, well-preserved stumps, snags, and/or living trees per site. At sites with less than seven visibly fire-scarred trees, we collected samples from every fire-scarred tree present. At sites with seven or more visibly fire-scarred trees, we collected samples from the trees or snags with the greatest number of visible fire scars. All fire scar samples used in our final fire chronologies were collected from ponderosa pine trees due to their better preservation of fire scars. We prepared the partial cross sections according to standard dendrochronological protocol and sanded them to enhance the visibility of ring boundaries [58], [59]. We visually crossdated the samples and identified fire dates by noting the years in which scars occurred. Crossdating accuracy was assessed using the program COFECHA [60] to ensure that each annual ring was correctly dated [58], [59] after measuring ring widths to the nearest 0.005 mm. We conservatively defined fire years as those in which at least two trees at a site were scarred, or in which a single fire scar was corroborated by a nearby, existing fire chronology. We used existing fire chronologies in lieu of creating new reconstructions for two sites (IMN, original site code: USIRC001; and BHR, original site code: USBGH001; [61], [52], [53]).

We used existing reconstructions of western spruce budworm outbreaks [15], in which outbreaks were identified through the comparison of ring-width series from host trees with a control chronology created using ring-width series from “non-host” ponderosa pine trees. A detailed explanation of these methods is reported in Flower et al. [15].

### Ethics statement

Our research sites were located on public land. Permits for field sampling were obtained where necessary from local National Forest Service and Bureau of Land Management offices.

### Analysis of climatic drivers of disturbances

At each site, we assessed the relationship between moisture availability and disturbance events with a superposed epoch analysis using dplR software [62], [63]. Superposed epoch analysis averages the climatic variable of interest at multiple temporal lags relative to each disturbance event, thereby creating a composite of the climatic conditions before and after disturbance events at each site. An 11-year window was used to assess the climate in the year of disturbances and in each of the 5 years preceding and following disturbances. We explored longer windows, but found no consistently significant associations beyond five years. Five years on either side of a disturbance event also seems to cover the most ecologically relevant climate associations as it includes the years most likely to alter fine surface fuels and influence annual trends in budworm populations. This procedure was repeated separately for fire dates and western spruce budworm outbreak initiation dates. For this analysis, outbreak initiation dates were defined as the first of at least two consecutive years in which an outbreak was recorded, preceded by two or more years without an outbreak (as detailed in [15]). We include climate both before and after outbreak initiation dates because insect outbreaks span multiple years, and may therefore be influenced by climate both before the outbreak begins (climatic conditions conducive to population growth leading up to outbreak conditions) and after the outbreak has started (conditions conducive to sustaining outbreak conditions). While climate after a fire cannot retroactively affect past fires, we include climate in post-fire years for symmetry with our insect-climate analysis.

We used Cook et al.'s (2004) gridded dendroclimatological reconstruction of the Palmer Drought Severity Index (PDSI) to represent moisture availability at our sites. The PDSI is a widely used measure of moisture stress [64] calculated from temperature, precipitation, and soil type. We chose the annual summer (June-August) PDSI from one of the four nearest grid cells to each site that had the highest Pearson's correlation with the host ring-width index (grid cells 44, 56, 57, 68, 69, 83, 84). In cases where PDSI at multiple grid cells were equally strongly correlated with the host-tree ring-width indices at that site, records from multiple grid cells were combined via simple averaging. We assessed the statistical significance using 1,000 Monte Carlo simulations to estimate bootstrapped confidence intervals (1-α = 95%). To meet the assumptions for the bootstrap test, we removed autocorrelation from the PDSI time series using an ARMA model of an order determined based on Akaike's Information Criterion.

### Synergism between disturbances

We analyzed the degree of synchrony between fires and three measures of western spruce budworm outbreaks (initiation, duration, and intensity). We used different analytical techniques for each measure: 1) modified one-dimensional bivariate Ripley's K-function analysis to assess synchrony between fire dates and outbreak initiation dates, 2) chi-square test of independence to test the relationship between fire occurrence and outbreak duration, and 3) superposed epoch analysis to quantify the association between fire occurrence and outbreak intensity. We analyzed synchrony prior to 1890 because fires were absent from all but one of our sites after that date.

First, we assessed the degree of synchrony between the timing of fires and the initiation of western spruce budworm outbreaks. We did this for temporal lags ranging from 0 years (i.e., synchrony during the year of event) to 50 years because the temporal association between defoliation and fire could theoretically occur over a broad window of time. We looked both for patterns of temporal clustering of disturbance events, which we refer to as synchrony, and patterns of disturbance events occurring further apart in time than would be expected by chance, which we refer to as asynchrony. We used a modified one-dimensional bivariate Ripley's K-function analysis (K1D v1.2; [65], [66], [67]) to determine whether western spruce budworm outbreak initiations and fires occurred closer to each other in time than would be expected by chance. We assessed synchrony at each site during the period common to both the fire and western spruce budworm outbreak records for that site. We transformed the results to the L function [67] for ease of interpretation; positive L function values indicate synchrony, negative values indicate asynchrony (i.e., events alternating through time), and values near zero indicate independence (i.e., no relationship) of the records being compared. The statistical significance of the results was assessed using 1000 randomized simulations in which the fire dates were shifted in a circular fashion relative to the western spruce budworm outbreak dates by adding a random number of years to each fire history record. This maintained the inherent multi-decadal patterns of disturbance dynamics in the randomized data.

Second, we assessed the association of fire occurrence and outbreak duration (the length of time a forest stand has been experiencing a western spruce budworm outbreak). We calculated the number of consecutive years of western spruce budworm outbreak preceding each fire date and binned them into four classes (0, 1–5, 6–10, and>10 cumulative outbreak years), with expected frequencies>5 in each class as required for robust estimation of the chi-square test [68]. We tested the hypothesis that there was no relationship between fire occurrence and outbreak duration with a chi-square test of independence (1-α = 95%). In this analysis, we created a contingency table to test for differences between the number of fires occurring in each class of outbreak duration with the expected number of fires that would occur in that class if fires occurred randomly with respect to outbreaks.

Third, we analyzed the association of fire occurrence and outbreak intensity (percent of trees in a stand that were experiencing a western spruce budworm outbreak), using superposed epoch analysis. This analysis, applied separately to each site, quantified the mean percentage of trees experiencing a western spruce budworm outbreak in each of the ten years preceding and following fire years and during fire years, and we assessed statistical significance using bootstrapping as described above.

## Results

### Disturbance histories

**Figure 2:**
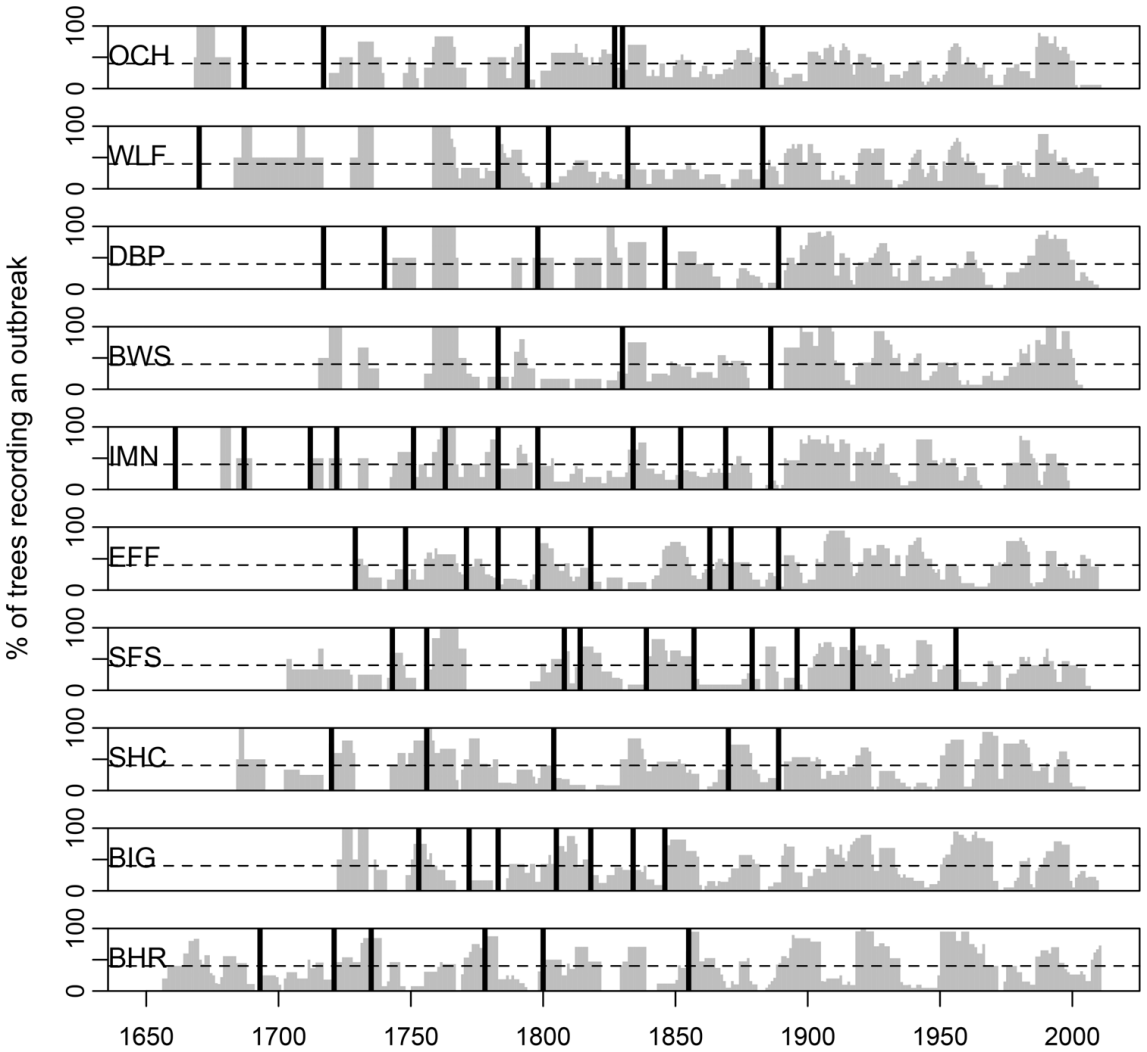
Tree-ring reconstructed chronologies of outbreaks of western spruce budworm (gray bars) and fire (vertical black lines). Dashed lines are the 40% threshold used to identify outbreak periods. The sites are arrayed from west (top) to east (bottom). See S1 Material for detailed disturbance dates.

Our eight new and two previously published fire chronologies have starting dates (i.e., date of first recorded fire) between 1618 and 1783 (Table 1; Fig. 2). Between 3 and 12 fires were recorded at each site (see S1 Material for fire dates). Before 1900, the average within-site-composite fire-return interval ranged from 16 to 53 years and averaged 34 years when pooled among sites. Only one site (SFS) recorded two fires during the early 20^th^ century, while the remaining sites recorded their last fire between 1846 and 1889. Seven of the ten sites recorded their last fire in the seven-year period between 1883 and 1889. There was a low level of synchrony of fires among sites, with only 12 of the 52 fire years recorded in at least 2 of our 10 sites.

The ten outbreak chronologies have start dates between 1640 and 1739 (Table 2; Fig. 2). Each site experienced between 8 and 16 outbreaks, averaging 11.6 outbreaks per site (see S1 Material for outbreak dates). The duration of individual outbreaks varied widely (4–40 years), but the average duration of outbreaks was fairly consistent among sites, ranging from 8 to 15 years among sites and 11.5 years across all sites. The quiescent period between individual outbreaks was highly variable (3–61 years). The average quiescent period varied from 10 to 23 years at individual sites, with an average quiescent period of 15.7 years across all sites. The length of both outbreaks and quiescent periods changed during the latter part of the record. The average outbreak duration increased from 10.3 years before 1890 to 13.2 years after 1889. The average length of quiescent periods decreased from 19.5 years before 1890 to 12.8 years after 1889.

**Table 2:** Western spruce budworm outbreak and fire reconstruction characteristics.

Site	Outbreak record length	No. of outbreaks	Average outbreak length	Average quiescent period length	Fire record length	No. of fires	Average return interval
OCH	1668–2010	15	10	13	1570–2010	8	39
WLF	1680–2010	11	8	22	1670–2010	5	53
DIX	1739–2009	8	13	19	1717–2009	5	43
BWS	1715–2010	12	10	15	1783–2010	3	52
IMN	1678–2009	9	15	23	1661–1993	12	21
EFF	1701–2010	11	11	15	1729–2010	9	20
SFS	1703–2009	12	10	12	1743–2009	10	22
SHC	1683–2009	12	12	13	1720–2009	5	42
BIG	1708–2009	10	14	15	1753–2009	7	16
BHR	1640–2010	16	12	10	1693–2003	6	32
**Average:**	**331 years**	**11.6**	**11.5**	**15.7**	**303 years**	**7**	**34**

Reconstructed outbreak record length (start date and end date), number of outbreaks reconstructed, average length of reconstructed outbreak periods, average length of reconstructed quiescent (non-outbreak) periods, reconstructed fire record length (start date and end date), number of fires reconstructed, average length return interval between fires (before 1890).

### Climatic drivers of disturbances

**Figure 3:**
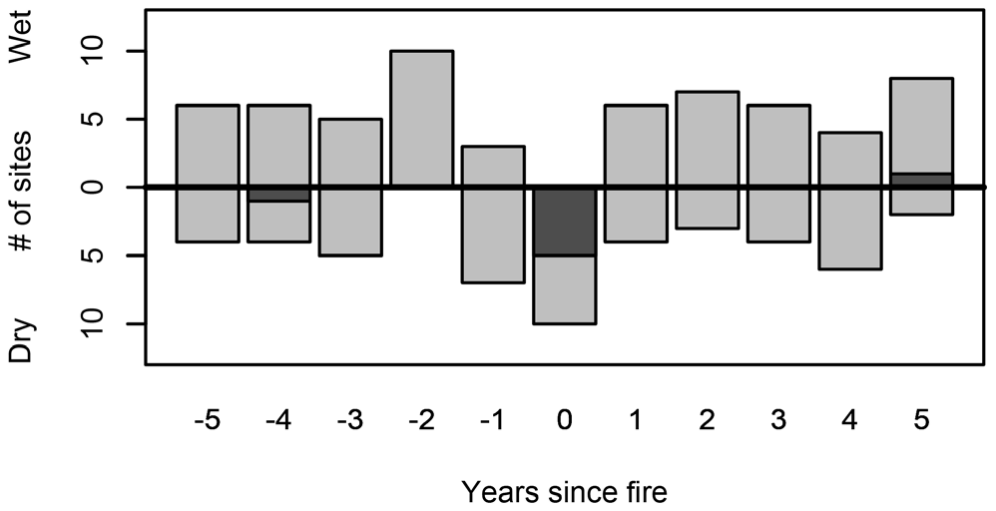
Summary of superposed epoch analyses conducted at each of our 10 study sites, indicating the direction of Palmer Drought Severity anomalies for an 11-year window centered on fire dates. Descending bars show the number of sites with a negative association with PDSI, ascending bars show the number of sites with a positive association with PDSI. Dark grey shading shows number of sites with significant at the 95% confidence interval anomalies.

**Figure 4:**
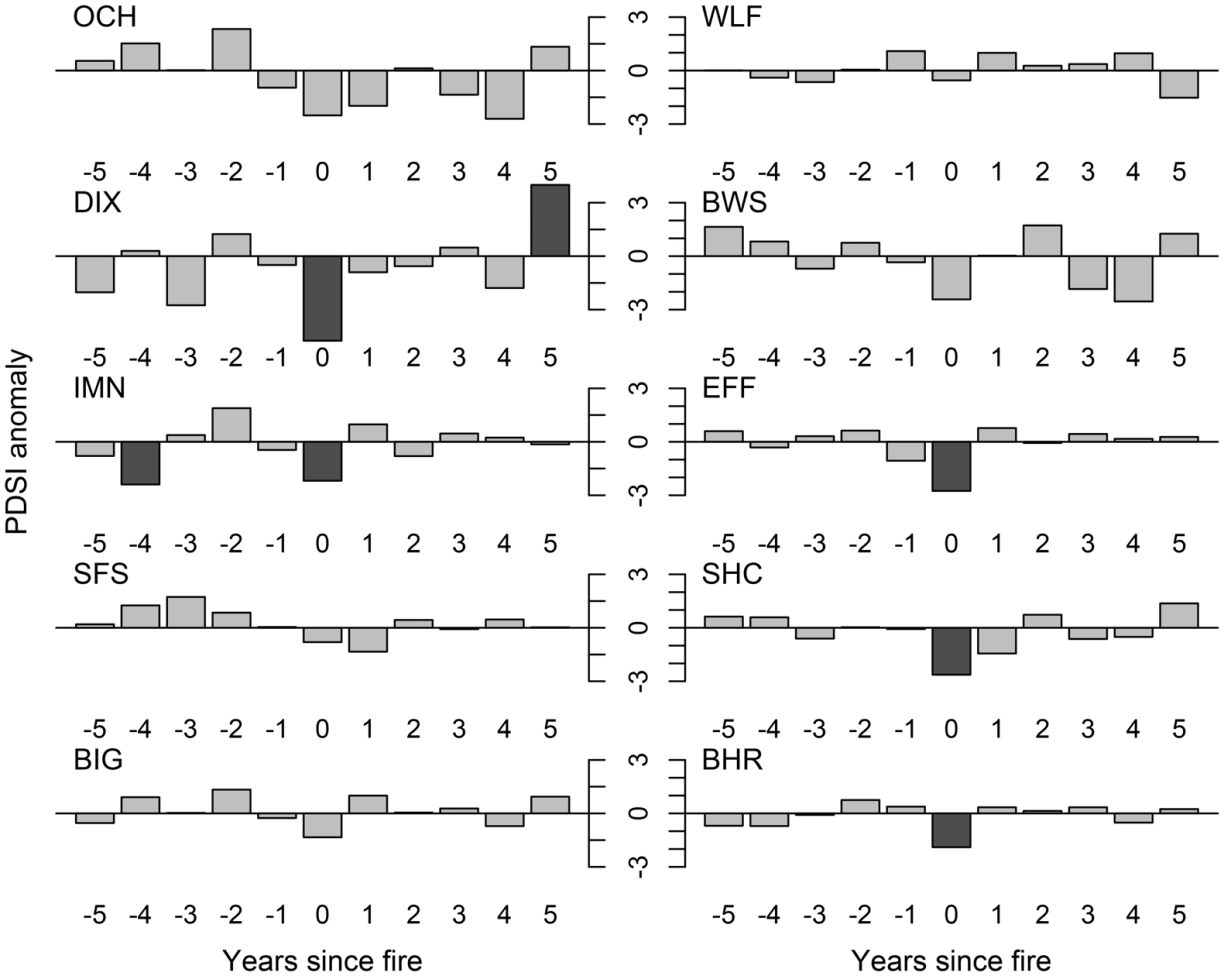
Superposed epoch analysis indicating the direction of Palmer Drought Severity anomalies for an 11-year window centered on fire dates at each of our 10 sites. Descending bars indicate a negative association with PDSI (i.e., droughty conditions), ascending bars indicate a positive association with PDSI (i.e., wetter conditions). Dark grey shading shows statistically significant (at the 95% confidence interval) anomalies.

**Figure 5:**
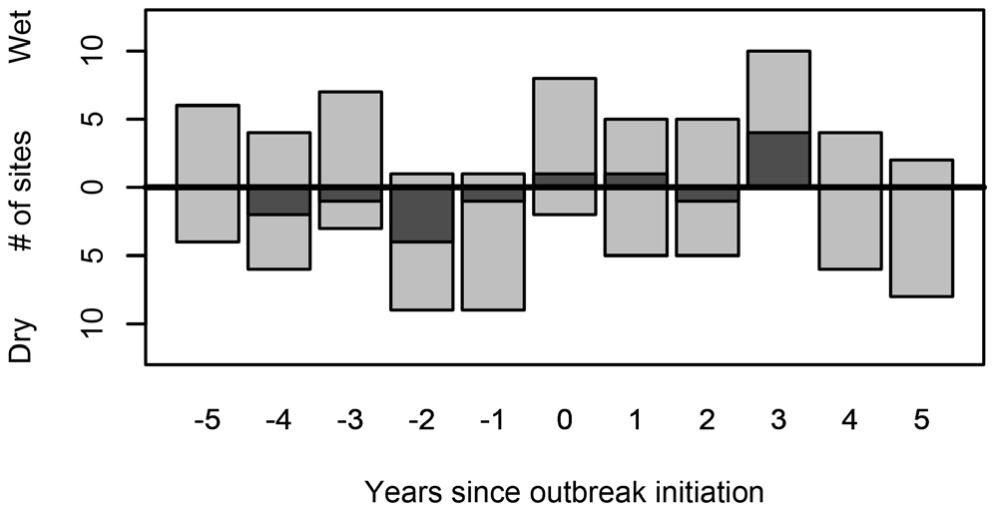
Summary of superposed epoch analysis indicating the direction of Palmer Drought Severity anomalies for an 11-year window centered on outbreak initiation dates. Descending bars show the number of sites with a negative association with PDSI (i.e., droughty conditions), ascending bars show the number of sites with a positive association with PDSI (i.e., wetter conditions). Dark grey shading shows number of sites with statistically significant (at the 95% confidence interval) anomalies.

**Figure 6:**
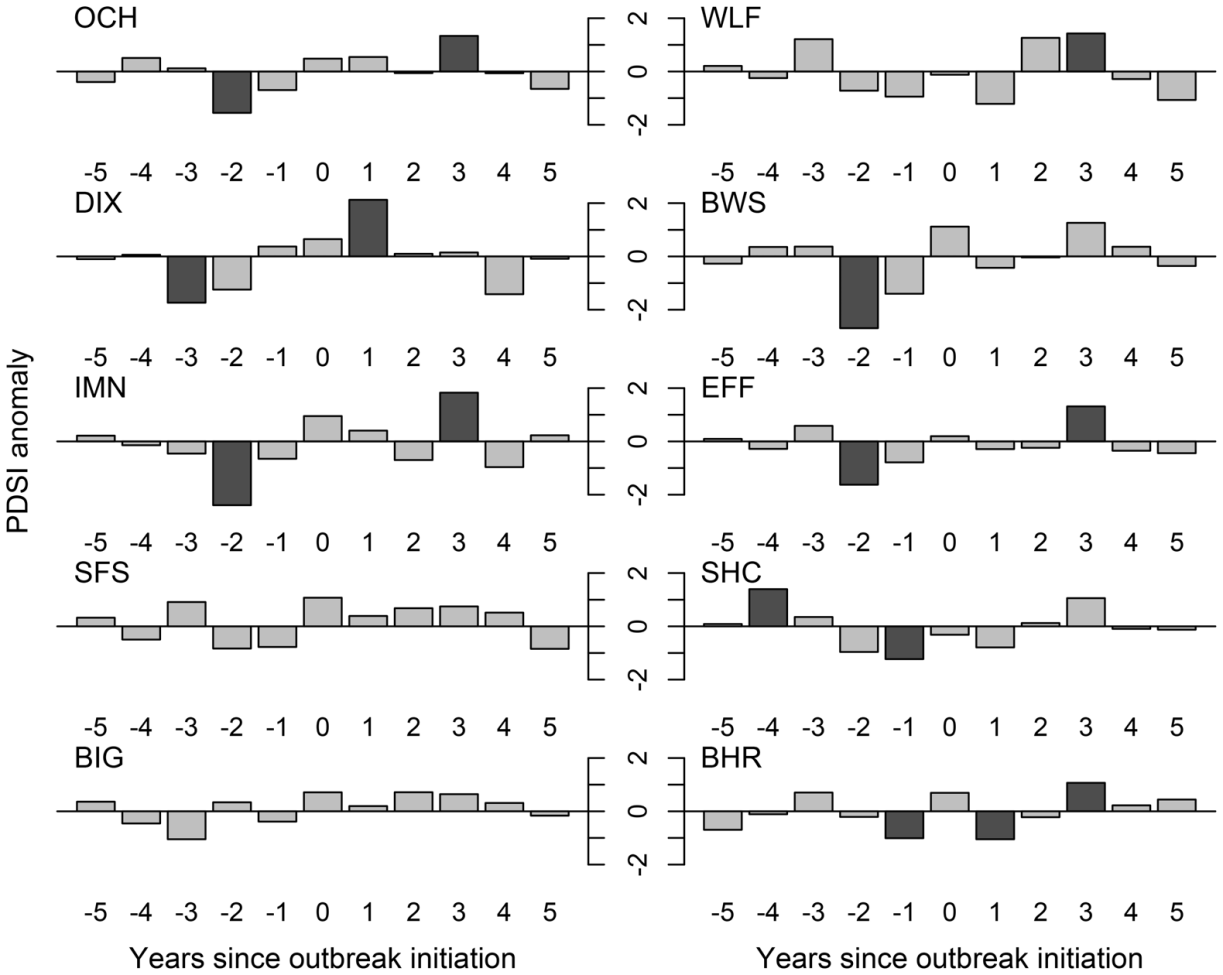
Superposed epoch analysis indicating the direction of Palmer Drought Severity anomalies for an 11-year window centered on outbreak initiation dates at each of our 10 sites. Descending bars indicate a negative association with PDSI (i.e., droughty conditions), ascending bars indicate a positive association with PDSI (i.e., wetter conditions). Dark grey shading shows statistically significant (at the 95% confidence interval) anomalies.

Fires tended to occur during years with low summer PDSI (i.e., periods of below average moisture availability). All ten sites experienced below-average PDSI during fire years, but the departure was significant at only half the sites (Fig. 3; Fig. 4). No significant pattern of climatic anomalies was apparent in the five years preceding fire dates. However, all ten sites experienced low magnitude, non-significant positive PDSI anomalies two years before fires. Western spruce budworm outbreaks typically initiated in times of transitional climatic conditions near the end of droughty periods. Western spruce budworm outbreak initiation dates were preceded by 2 to 4 years of warm-dry conditions at all sites (Fig. 5; Fig. 6). All ten sites experienced negative PDSI anomalies during at least two of the four years preceding outbreak initiation. Seven sites experienced one or more years of significantly negative PDSI anomalies during the four-year period preceding outbreak initiation. Outbreak initiations were typically followed by positive PDSI anomalies. All ten sites experienced positive PDSI anomalies during two or more years during the four year-window starting in the year of outbreak initiation. Six sites experienced at least one year of statistically significant positive PDSI anomalies during this four-year window.

### Synergism between disturbances

**Figure 7:**
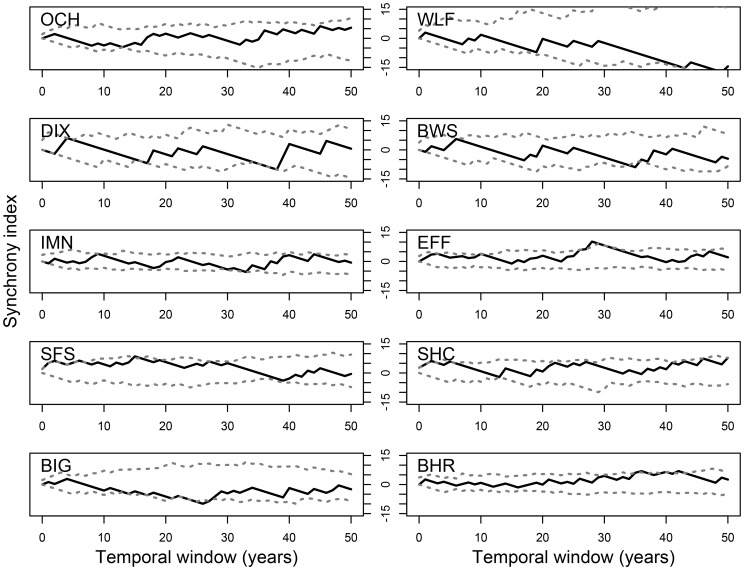
Synchrony of fire occurrence and the initiation dates of western spruce budworm outbreaks, analyzed using the modified Ripley's K-function at each site. Synchrony index presented as L-function for ease of interpretation. Positive L function values indicate synchrony, negative values indicate asynchrony, and values near zero indicate independence of the records being compared. Dashed grey lines are 95% confidence intervals.

**Figure 8:**
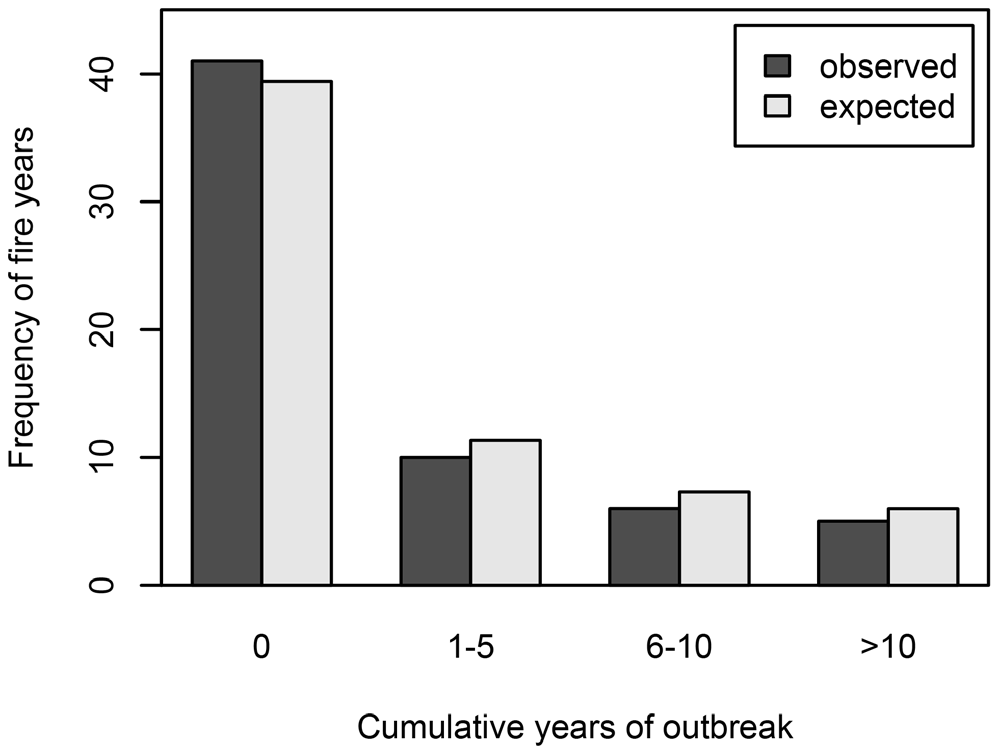
Distribution of fire years by outbreak duration (cumulative number of years of western spruce budworm outbreak). Expected distribution is based on fires occurring randomly with respect to the timing of outbreaks.

**Figure 9:**
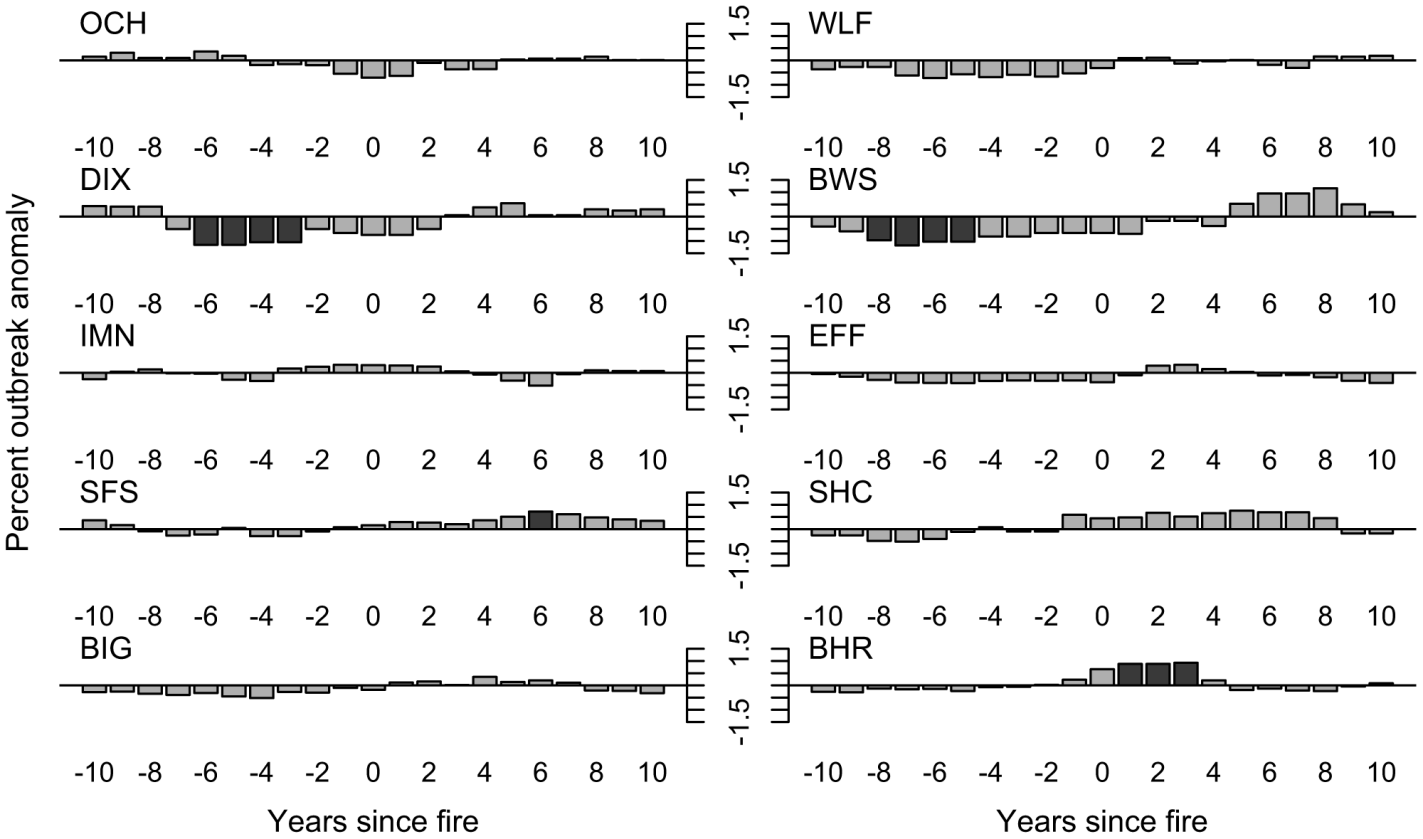
Superposed epoch analyses of the percent of trees recording a western spruce budworm infestation for a 21-year window centered on fire dates (see Fig. 2). Descending bars indicate a below average percent of trees recording infestation (i.e., less infestation), ascending bars indicate an above average percent of trees recording infestation (i.e., more infestation). Dark grey shading shows statistically significant (at the 95% confidence interval) anomalies.

At our sites before 1890, fire was not significantly associated with any of our three measures of western spruce budworm outbreaks: initiation, duration, or intensity. First, the modified Ripley's K-function analysis revealed no consistent pattern of synchrony or asynchrony between fire occurrence and outbreak initiation at any of the sites (Fig. 7). Second, although nearly two-thirds of all fire years occurred during non-outbreak conditions (Fig. 8), fires were no more likely to occur during non-outbreak years than would be expected by chance (χ^2^ = 0.5646, df = 3, *P* = 0.9045). Third, there was a fairly consistent trend towards less intense western spruce budworm infestations in the ten years preceding fires, but the anomalies were generally of a small magnitude and rarely statistically significant, with only two sites showing a significant relationship at any antecedent lag (Fig. 9).

## Discussion

In general, our sites experienced disturbance frequencies of fire and western spruce budworm outbreaks broadly similar to those found by other studies in the region. Unlike previous studies, our disturbance histories were obtained from the same stand, which allowed us to assess their influence on each other. The discussion below summarizes the disturbance histories, the relationship of each disturbance type with moisture availability, and the lack of inter-disturbance synergism apparent in our records.

### Disturbance histories

The ten forest stands we studied have sustained numerous western spruce budworm outbreaks and fires over the last three centuries. Mean fire-return intervals at our sites are similar to those reported elsewhere for mixed-conifer forests in the interior Pacific Northwest [47], [6], [48]. For example, the mean fire-return intervals at our sites (range: 16–53 years; plot size: <0.01^2^ km) do not differ greatly from those reported for sites in the Blue Mountains of northeastern Oregon (range: 10–49 years; study plots: 0.0005^2^ m; [48]) and for the southern Bitterroot Valley in western Montana (range: 6–19 years; study plots: <3.2^2^ km; [47]), although differences in study plot size make direct comparisons difficult. The mean duration of western spruce budworm outbreaks at individual stands (12 years) is very close to the mean duration reported for similar studies in in northern New Mexico (11 years; [12]), northeastern Oregon (15 years; [13]), and southern British Columbia (12 years; [51]). These similarities reveal regional coherence of mixed-conifer disturbance regimes over a large geographic extent. This coherence shows that our sites can be considered reasonably representative of the range of disturbance regimes in mixed-conifer forests in the interior Pacific Northwest.

The low- or mixed-severity fires that occurred fairly frequently in these forests prior to the 20^th^ century became rare or entirely absent during the 20^th^ century. All but one of our sites recorded no fires after 1890 despite a robust 20^th^ century tree-ring record. About two-thirds of our sites recorded their last fire during the 1880s, suggesting that the 1890s mark the onset of widespread fire exclusion in this region, consistent with other estimates of the timing of fire exclusion in the region [49], [43]. The shift towards lower frequency, possibly higher severity fire regimes has been identified in many Pacific Northwest forest ecosystems [9] and has been attributed to the onset of extensive logging, grazing, and active fire suppression [9], [39]. The early onset of fire exclusion recorded at our sites suggests that grazing may have been highly influential. Grazing became an important factor in shaping forest characteristics decades before the technology, infrastructure, and population density required for effective fire suppression and extensive logging existed. Euro-American settlers began grazing large numbers of livestock in Pacific Northwest forests during the mid-19th century and most suitable forests were being heavily grazed by the late 1800s [69], [70], [71]. Grazing livestock caused a decrease in fire frequency in many forests by severely reducing herbaceous biomass and fine fuels [69], [70], [71].

At our ten sites, western spruce budworm outbreaks became more frequent and longer lasting during the 20^th^ century than during previous centuries. In a separate analysis, we also found that outbreaks became more synchronous across our transect after the late 19^th^ century [15]. Similar 20^th^ century increases in the severity of growth reduction [42], [13], and the duration [42], frequency [13], and regional synchrony [11], [12] of western spruce budworm outbreaks have been reported in other studies. These changes are often attributed primarily to changing land-use practices, particularly the widespread effects of fire exclusion. Fire exclusion has led to a shift from patchy, heterogeneous landscapes with many open stands dominated by shade-intolerant, fire-resistant species towards homogeneous landscapes characterized by dense stands of shade-tolerant, fire-sensitive tree species in many areas [69], [9], [39]. These changes in forest structure and composition have created ideal conditions for more widespread and severe defoliation by the western spruce budworm by increasing the extent and homogeneity of densely stocked forests composed of tree species favored by the insect [38], [7], [39], [40]. Additionally, the increased density of understory trees has created a multi-layered forest structure that enables caterpillars to easily disperse downwards through the canopy layers with a low risk of failing to find suitable food or falling to the forest floor [38], [40]. The changes in western spruce budworm outbreak patterns at our sites since the late 1800s support the hypothesis that fire plays an important role in governing long-term outbreak characteristics at the landscape scale through its effects on the quantity and distribution of host trees.

### Climatic drivers of disturbances

Climate was a significant driver of both fire and western spruce budworm outbreak dynamics. Fires tended to occur during drought years at all sites. We found no statistically significant association with moisture availability during preceding years. The lack of statistical significance and typically small magnitude of anomalies in prior years indicates that moisture availability during preceding years is generally of less importance than conditions in the year of fire occurrence. This suggests that fuel moisture is a more significant limiting factor than fuel availability in these forests. Increased moisture in prior years is an important driver of fire occurrence in many relatively xeric forests where fire spread is constrained by inadequate surface fuel loads [22], [74], [45]. In xeric forests, wet years correspond with increased production of fine fuels and thus increase the likelihood of fires occurring in the following year. In contrast, more mesic mixed-conifer forests typically have adequate fuel loads to carry fire in any given year and therefore do not exhibit the same lagged relationship between climate and fire dynamics [22], [74], [45]. We found a consistent association between moisture availability and the initiation of western spruce budworm outbreaks, as more fully discussed in Flower et al. [15]. Outbreaks tended to occur during times of transitional climatic conditions, near the end of droughty periods.

### Synergism between disturbances

We found no evidence of a consistent relationship between the timing of fires and western spruce budworm outbreaks. Although both disturbances were associated with reduced moisture availability, they occurred randomly in time relative to each other. This lack of association may be explained by two factors. First, each disturbance type is associated with slightly different climatic events, with western spruce budworm outbreak initiations associated with the ends of droughty periods and fires simply associated with single warm-dry years. The two disturbance types therefore respond to climatic variability over different temporal scales. It appears that the cumulative effect of multiple years of climate conditions can predispose a stand to western spruce budworm outbreaks. In contrast, fire ignitions occur stochastically, and the fuels that drive fire spread and intensity can equilibrate (i.e., desiccate or moisten) in as little as a few hours or days [72], [73]. Second, fire dynamics in these forests are not strongly fuel limited, as evidenced by the lack of association between fire and antecedent climate. Rather, fuel moisture and ignition sources appear to be the dominant limiting factors on fire occurrence in these forests. There is a well-established positive association between relatively warm, warm-dry years and the probability of large fire occurrence [74], increased area burned [45], and regional fire synchrony [49], [50] in the interior Pacific Northwest. We propose that any effect of defoliation events on fuel availability is therefore likely masked by the overriding influence of climatic variability on fire risk.

Climatic variability can similarly complicate interpretations of the interactions between fires and past bark beetle outbreaks [26]. Weather appears to exert much stronger control on fire behavior than previous insect outbreak activity [75], [33], [76]. Bark beetle outbreaks several years prior to a fire have been found to have no influence [77], [75] or a damping effect [32], [33] on fire risk. Temporal dynamics of insect outbreak-induced fuel changes can also complicate the picture [28], [78]. Several years after bark beetle outbreaks, their primary impact appears to be a reduction in canopy fires due to decreased canopy fuels [33]. Conversely, the probability of canopy fires may actually be increased in the early stages of an outbreak due to decreased fuel moisture [29], [78]. The net effect of bark beetle activity is thus highly dependent on time-since-outbreak [28], [33].

The net effect of defoliation on fuel availability remains uncertain. Although we found no synchrony or asynchrony between the two disturbance types in terms of probability of occurrence, there is growing evidence that western spruce budworm defoliation may actually decrease subsequent fire risk [24], intensity [37], or size [25]. The most visually obvious effect of defoliation is an increase in fine and coarse surface fuels through the accumulation of dead needles and wood, and, to a lesser extent, a short-term increase in dry canopy fuels in the form of partially consumed dead needles that remain in the canopy [79], [80]. However, dry needles typically remain in the canopy for less than a year [79]. This decrease in foliar moisture affects a small portion of total foliar mass during a brief temporal window and is likely a much weaker effect than the overall decrease in canopy fuels that occurs as needles are consumed. Mortality of saplings may have a longer-term effect on fire behavior via a reduction in ladder fuels [81]. The net effect of defoliation may therefore be a damping of future fire severity through a reduction in the probability of crown fire [37]. Opening of forest canopies through defoliation may also encourage the growth of herbaceous understory plants, which shade the forest floor and thus increase surface fuel moisture [32], [24].

The fact that we did not detect the negative association between western spruce budworm defoliation and subsequent fire risk reported in previous studies [24], [25] and detected in simulation modeling [37] may be due to differences in the spatial and temporal scale of our studies. Our analyses were focused on stand-scale dynamics, so we could not assess the spatial extent of disturbance events. Our records span three centuries, while previous studies were limited to 20–30 years at the end of the 20^th^ century [24], [25]. A direct comparison of the results of these studies is constrained by the fact that they focused on different fire characteristics. Our study and Lynch and Moorcroft's [24] work analyzed the occurrence of disturbance events, while Preisler et al. [25] assessed defoliation impacts on fire extent, and Cohn et al. [37] quantified defoliation impacts on fire intensity. Lynch and Moorcroft [24] found that fires were less likely to occur in the 7 years following an outbreak and outbreaks were less likely to occur in the 6 years following a fire, but this relationship was highly dependent on how close the outbreaks and fires occurred in space and time. For instance, they reported an increased probability of western spruce budworm outbreak in the same year in which fire occurred for areas within 1 km of the fire, but reduced probability at other distances and temporal lags. Preisler et al. [25] found a statistically significant negative relationship between the size of western spruce budworm outbreaks and the size of subsequent fires for relatively large outbreaks, but no significant relationship for outbreaks less than 100 hectares in area. While these studies suggest a negative correlation across broad regions, our study showed no correlation over 300 years in individual stands.

Our site-selection strategy targeted sites with numerous old, living trees, which necessarily excluded sites with only young trees. Thus, we did not samples stands that had experienced a stand-wide high-severity fire during the last 250-300 years. Our fire-reconstruction method gave us annually-resolved records of low-severity fires, but could not capture the full range of fire severities responsible for the complex mosaic of tree ages typically seen at the landscape scale. Interpretation of our results must therefore be limited to forest stands in mixed- or low-severity fire regimes. However, the fact that we found multiple stands across a broad transect that had experienced numerous fires preceded by frequent, sometimes multi-decadal, western spruce budworm outbreaks suggests that the cumulative effect of these two disturbance types helps to maintain a low-severity disturbance regime. It is possible that these disturbance types create a self-regulating system wherein defoliation increases the likelihood of Douglas-fir and true fir trees surviving subsequent fires via a reduction in ladder fuels and canopy fuels, while fires in turn reduce the severity of subsequent western spruce budworm outbreaks by reducing the density of host trees, particularly in the understory. This synergistic damping effect corresponds well with the changes in outbreak characteristics we identified in the period following fire exclusion. We propose that synergy between the two disturbance types has altered disturbance severity, but not their probability of occurrence, over the last three centuries.

Our analysis of disturbance interactions was limited to the pre-1890 period in which fires occurred at our sites. The alteration of forest composition and structure brought about by changing land use and climate during the 20^th^ century must therefore be kept in mind when applying our interpretation of these results to modern forests. The shift towards denser forests, greater prevalence of shade-tolerant tree species, and increased ladder fuels observed in many regions may have consequences for disturbance interaction dynamics. However, the consistency of our results among all ten sites and the fact that our records contain a number of multi-decadal periods in which no fires were recorded indicates that the independence of fires and defoliation events is stable across a wide range of fuel conditions and forest compositions.

Temperature has increased across the interior Pacific Northwest over the 20^th^ century, and further increases in temperature are projected for the 21st century [82]. Projections of changes in precipitation are less certain, but a decrease in summer precipitation appears likely for this region [82]. Our results therefore support previous projections of increased fire [44], [83], [84], [43] and increased western spruce budworm outbreaks in some areas [85], [15], but also indicate that these disturbances will increase largely independently of each other in response to climate.

## Conclusion

We reconstructed multi-century records of western spruce budworm outbreaks and fires at ten sites in the interior Pacific Northwest. This is, to the best of our knowledge, the first analysis of long-term interactions between western spruce budworm outbreaks and fires. Our sites experienced frequent, long-lasting defoliation events and repeated fires. Drought was associated with the occurrence of both disturbance types. However, fires tended to occur during individual warm-dry years while western spruce budworm outbreaks tended to start near the end of droughty periods. Prior to 1890, no consistent relationship was apparent in the timing of the two disturbance types. Defoliation events appear to have no discernible impact on subsequent fire risk. If the production of fine and coarse fuels during defoliation events had any impact on fire risk, it was too subtle to detect given the overriding influence of climatic variability. After approximately 1890, fires were largely absent from these sites and western spruce budworm outbreaks became longer-lasting, more frequent, and more synchronous. This reveals a subtle synergistic relationship between the two disturbance types that influences their severity, but not probability of occurrence, over long time scales. Although budworm outbreaks and fire are currently occurring on a landscape that has little historical analog, our records show no precedent that future budworm outbreaks will increase fire risk.

## Supporting Information

S1 Material **Results from disturbance history reconstructions.**
(XLSX)
